# A High-Performance Cr_2_O_3_/CaCO_3_ Nanocomposite Catalyst for Rapid Hydrogen Generation from NaBH_4_

**DOI:** 10.3390/nano14040333

**Published:** 2024-02-08

**Authors:** Majed Alshammari, Khulaif Alshammari, Sultan Alhassan, Alhulw H. Alshammari, Turki Alotaibi, Satam Alotibi, Ali Ismael, Taha Abdel Mohaymen Taha

**Affiliations:** 1 Physics Department, College of Science, Jouf University, P.O. Box 2014, Sakaka 72388, Saudi Arabia; knnalshammari@ju.edu.sa (K.A.); ssalhassan@ju.edu.sa (S.A.); ahalshammari@ju.edu.sa (A.H.A.); tbotaibi@ju.edu.sa (T.A.); themaida@ju.edu.sa (T.A.M.T.); 2 Department of Physics, College of Science and Humanities in Al-Kharj, Prince Sattam bin Abdulaziz University, Al-Kharj 11942, Saudi Arabia; sf.alotibi@psau.edu.sa; 3 Physics Department, Lancaster University, Lancaster LA1 4YB, UK; k.ismael@lancaster.ac.uk

**Keywords:** Cr_2_O_3_ nanoparticles, hydrogen energy, catalyst, band gap

## Abstract

This study aims to prepare new nanocomposites consisting of Cr_2_O_3_/CaCO_3_ as a catalyst for improved hydrogen production from NaBH_4_ methanolysis. The new nanocomposite possesses nanoparticles with the compositional formula Cr_2−x_Ca_x_O_3_ (x = 0, 0.3, and 0.6). These samples were prepared using the sol-gel method, which comprises gelatin fuel. The structure of the new composites was studied using X-ray diffraction (XRD), Fourier transform infrared spectroscopy (FTIR), Raman spectroscopy, environmental scanning electron microscopy (ESEM), and X-ray spectroscopy (XPS). The XRD data showed the rhombohedral crystallinity of the studied samples, and the average crystal size was 25 nm. The FTIR measurements represented the absorption bands of Cr_2_O_3_ and CaO. The ESEM micrographs of the Cr_2_O_3_ showed the spherical shape of the Cr_2_O_3_ nanoparticles. The XPS measurements proved the desired oxidation states of the Cr_2−x_Ca_x_O_3_ nanoparticles. The optical band gap of Cr_2_O_3_ is 3.0 eV, and calcium doping causes a reduction to 2.5 and 1.3 eV at 15.0 and 30.0% doping ratios. The methanolysis of NaBH_4_ involved accelerated H_2_ production when using Cr_2−x_Ca_x_O_3_ as a catalyst. Furthermore, the Cr_1.7_Ca_0.3_O_3_ catalyst had the highest hydrogen generation rate, with a value of 12,750 mL/g/min.

## 1. Introduction

The world is currently facing severe environmental problems, one of which is carbon pollution (CP). CP is primarily caused by unclean energy sources such as fossil fuels, coal, oil, natural gas, and nuclear power. These forms of energy release pollutants into the air and water, which are harmful to the environment. In order to mitigate these problems, renewable energy is indeed considered one of the most critical solutions to environmental problems. Renewable energy is generated from natural resources, such as sunlight, wind, water, and geothermal heat. The greatest benefits of renewable energy are it being clean (carbon-free) and sustainable. As the world continues to seek alternative energy sources, hydrogen gas (H_2_) is a new energy source. H_2_ has attractive prospects because it is a method with promise in terms of its energy carriers [1]. Although hydrogen is an environmentally friendly and sustainable fuel, the major challenges surrounding hydrogen-based energy are its production, storage, and delivery [1,2]. One of the most well-liked methods for producing hydrogen is the hydrolysis reaction due to its high efficiency [3,4]. Hydrogen storage has occurred in many compounds, such as sodium borohydride [5], ammonia borane [6], hydrazine hydrate [7], water [8], methanol [9], and magnesium hydrides [10]. These compounds have become the most promising for on-site hydrogen production.

Sodium borohydride (NaBH_4_) is a promising material for hydrogen generation due to its hydrogen capacity of approximately 10.8 wt. % [11]. NaBH_4_ has good stability, making it easy to transport [12]. Thus, NaBH_4_ is a promising compound for hydrogen storage and its production according to a methanolysis reaction [13]. When using the hydrolyzing procedure for NaBH_4_, hydrogen can be liberated as $NaBH_{2}\left(s\right)+2H_{2}O\left(1\right)\to NaBO_{2}\left(aq\right)+4H_{2}$. The standard enthalpy change in a reaction is $∆H^{\Theta }$ = −216.7 kJ/mol of sodium borohydride [14,15]. H_2_ can be generated from a stabilized NaBH_4_ solution through dominant hydrolysis. The hydrolysis of NaBH_4_ is a standard method used to accelerate H_2_ production [16,17]. Indeed, catalysts play a critical role in developing nanomaterials using chemical reactions such as decomposition reactions [18]. These catalysts possess unique properties that make them ideal for various applications [19,20,21,22]. The catalyst can be used to enhance the efficiency of hydrogen production by lowering the activation energy of the reaction. Fernandes et. al. estimated the value of the activation energy to be 13 kJ/mol; this activation energy is reduced by a factor of 5 using only methanol solutions [22]. The results of a hydrolysis reaction in producing H_2_ can be significantly influenced by using catalysts to accelerate the reaction. The hydrolysis of NaBH_4_ demands an appropriate catalyst to control the H_2_ production. Therefore, the generation of hydrogen, the development of efficient catalysts, and cost-effectiveness are critical issues [23].

It is well known that nanomaterials (NMs) possess unique characteristics that make them highly desirable for a wide range of applications, including electronics [24], healthcare [25], manufacturing [26], and energy [27]. In order to achieve the distinct properties required for H_2_ production, it is crucial to consider the surface area and redox properties of these NMs. They are extensively utilized in diverse applications such as catalysis [28], sensors [29], and solar cells [30]. Various materials have been explored as effective catalysts for H_2_ production, including metal oxides [15], metal sulfides [31], and metal carbonyls [32]. For example, extensive research has been conducted on the impact of catalysts such as metal oxides, including MgO [33], CaO [34], Fe_2_O_3_ [35], CuO [36], and Ag_2_O [37], on the catalytic hydrolysis of NaBH_4_. A study by Neslihan Er et al. [38] focused on hydrogen generation from NaBH_4_ using a Co/CuO–NiO–Al_2_O_3_ catalyst. The researchers discovered that the catalyst exhibited a significant reaction activity (HGR) of 2067.2 mL min g^−1^ at room temperature, with an activation energy of 31.59 kJ mol^−1^. Additionally, F. Wang et al. reported on the hydrogen generation capabilities of composite catalysts. In their studies, they synthesized a series of dandelion-like Co-P/CNT-Ni foam catalysts and studied their catalytic characteristics for hydrogen production from NaBH_4_ methanolysis. Furthermore, their results showed that the Co-P/CNT-Ni foam catalysts retained their dandelion-like structure and achieved a maximum hydrogen generation rate of 2430 mL min^−1^ g^−1^ at 25 °C. Moreover, the Co-P/CNT-Ni foam catalysts exhibited am excellent cycling performance and a low activation energy (49.94 kJ mol^−1^) for the methanolysis of sodium borohydride. [39]. Another study investigated the effect of solvents on the hydrogen generation activity of Ru-Co nanoparticles during the catalytic dehydrogenation of sodium borohydride (NaBH_4_). They synthesized and characterized the nano-sized catalyst using various techniques, including TEM analysis. They also conducted kinetic investigations using water or methanol as the dehydrogenation mediums and analyzed the results using power law reaction kinetics and Arrhenius plots. Their results revealed that the use of water as a solvent resulted in a higher hydrogen generation activity than methanol due to the higher surface area of the catalyst in water [40]. In addition, a previous study on hydrogen generation from the methanolysis of NaBH_4_ over a Co/Al_2_O_3_ catalyst was conducted. The study found that the Co/Al_2_O_3_ catalyst, prepared following the impregnation–chemical reduction method, exhibited an excellent catalytic activity for the methanolysis of NaBH_4_ with a desirable hydrogen generation rate, even at low environmental temperatures. The byproduct of the methanolysis reaction was analyzed, and the characterization revealed that methanol could be effectively recovered. They concluded that the catalytic activity of Co/Al_2_O_3_ could be further improved for NaBH_4_ methanolysis using an appropriate calcination treatment, which played a crucial role in the process. [41]. Furthermore, several previous studies have shown that noble-metal-based catalysts such as Pd, Pt, and Rh exhibit a good catalytic activity and robust stability for hydrogen generation [42,43,44,45]. On the other hand, their application in industry is restricted by their high costs and self-agglomeration. Thus, a suitable catalyst must solve these problems. Besides this, the preparation of nanocomposites can be carried out using electrodeposition [46], hydrothermal [47], sol-gel [48], chemical bath deposition [49], and polycondensation methods [50]. The simplest method many researchers use is the polycondensation method, which is a valuable technique for preparing nanomaterials [51]. In this paper, we have synthesized calcium/Cr_2_O_3_ for hydrogen production. Therefore, chromium (III) oxide (Cr_2_O_3_) is a promising material due to its low cost; wide utilization in applications, including solar energy collectors; its non-reciprocal optical properties; etc. [52,53,54,55,56,57]. In addition, Cr_2_O_3_ is studied to accelerate NaBH_4_ hydrolysis reactions. A previous study investigated the effect of concentrations of Cr (VI) and Cr (III) precipitation treated using NaBH_4_. Their results showed that oxalate can significantly enhance NaBH_4_’s reduction of Cr (VI). They also investigated the effects of oxalate Cr (VI) reduction and Cr (III) precipitation, which have been proven to aid the application of NaBH_4_ in industrial wastewater treatment [58]. Furthermore, calcium carbonate is known as an inorganic material with the chemical formula CaCO_3_. CaCO_3_ is a versatile chemical compound with a wide range of applications, including construction [59], agriculture [60], water treatment [61], and cosmetics [62]. CaCO_3_ can be used as a catalyst for H_2_ production due to its inexpensive cost and simple preparation [61]. Moreover, CaCO_3_ is abundant and a primary component of limestone, chalk, and eggshells [63,64]. Several studies have been conducted on the use of CaCO_3_. For example, Milad Piri et al. [65] studied hydrogen evolution on CaCO_3_ at different temperatures under electrochemical precipitation. The results indicated that the rate of hydrogen evolution increased with both the contribution of water reduction and the rate of water reaction. Moreover, Deheri et. al. [66] reported that hydrogen or methane content enhancement can be enhanced using CaO_2_ + CaCO_3_ and NaOH. They observed that the produced gas increased to 33.85% using CaO_2_ + CaCO_3_, and the methane content improved to 67.24% using NaOH as an alkali material. Taufiq-Yap et al. [67] also reported a high hydrogen production using a mixture of ZnO and Ni with CaO, achieving a value of 105.7 mmol mL^−1^. A new catalyst featuring Co_2_P nanoparticles strongly coupled with P-modified NiMoO_4_ nanorods grown on nickel foam and encapsulated by a carbon layer was developed for the hydrogen evolution reaction (HER) [68]. This combination offers abundant heterogeneous interfaces, strong electronic interactions, and optimized reaction kinetics. The catalyst showed an excellent HER performance with low overpotentials (105 mV in acid, 107 mV in base) for a 100 mA/cm^−2^ current density. A novel alternating electrodeposition strategy was used to synthesize a (P-Co/Ni_3_P)_A3_/NF electrode with excellent bifunctional activity for both the hydrogen evolution reaction (HER) and the alternating hydrazine oxidation reaction (HzOR) [69]. This research offered a promising strategy to significantly reduce the energy consumption of water electrolysis and pave the way for efficient grid-scale hydrogen production. The study revealed low potentials for both the HER and HzOR. The constructed two-electrode electrolyzer also shows impressive results, requiring very low cell voltages to achieve high current densities.

In this study, we aim to prepare a new nanocomposite using a simple and low-cost method for H_2_ production. The nanocomposite, Cr_2_O_3_-doped CaCO_3_, functions as a catalyst for generating H_2_ through the methanolysis of NaBH_4_. We investigated the structure and characterization of Cr_2_O_3_@CaCO_3_ using XRD, FTIR, and ESEM techniques. Furthermore, the optical absorption properties were studied in the UV-V wavelength range of 200 to 1000 nm. Finally, we studied and developed the catalytic activity of Cr_2_O_3_@CaCO_3_ for the methanolysis of NaBH_4_ at room temperature to generate H_2_.

## 2. Experimental Methods

The chemicals used in this work to prepare the Cr_2−x_Ca_x_O_3_ (x = 0, 0.3, and 0.6) nanoparticles were chromium (III) nitrate (nonahydrate) at 97%, calcium nitrate tetrahydrate at 98%, and gelatin powder provided by Loba Chemie, India. The total mass of Cr (NO_3_)_3_·9H_2_O and Ca (NO_3_)_2_·4H_2_O was twice the mass of gelatin. Accordingly, stoichiometric ratios of the materials used in the synthesis were dissolved in deionized water at 80 °C. The solution mixture of the gelatin and metal nitrates was stirred for 2 h at 80 °C. After that, the solutions were transferred into an electric oven for 4 h at 250 °C. During this process, water evaporated, and gases were released according to the combustion technique. Finally, the resulting samples were ground and further calcinated at 500 °C for 4 h to enhance their crystallinity. X-ray diffraction (XRD) can be used to determine the crystal structure of nanoparticles. The Shimadzu XRD-7000 has a high-resolution detector that can provide accurate measurements of diffraction peaks and was thus used to analyze the crystal structure of the Cr_2−x_Ca_x_O_3_ nanoparticles. The Shimadzu IRTracer-100 is a Fourier transform infrared (FTIR) spectrometer that was used to take attenuated total reflection (ATR) measurements of the nanoparticles. The powerful Quattro ESEM tool was used to study the microstructure properties of the nanoparticles. The surface area and pore size analyzer NOVA 4200e was used to obtain the nitrogen isotherm loops of the prepared nanoparticles. Thermo Fisher Scientific’s (Waltham, MA, USA) K-Alpha XPS spectrometer is a high-performance XPS system that was used to study the chemical composition and electronic structure of the prepared nanoparticle surfaces. The Thermo Scientific Evolution 201 benchtop UV-Vis spectrophotometer was used to measure the absorption spectra of the obtained nanoparticles.

The methanolysis reaction of NaBH_4_ in methanol is a relatively slow reaction. However, it can be accelerated according to the addition of a catalyst. A catalyst is a substance that speeds up a chemical reaction without being consumed in the reaction. Therefore, Cr_2−x_Ca_x_O_3_ nanoparticles were used as a catalyst for the methanolysis reaction of NaBH_4_ in methanol to produce hydrogen gas. The reaction was completed inside a glass vessel attached to a water displacement system. The catalyst material (0.02 g) was mixed with 0.25 g of NaBH_4_ and then poured into the vessel. Thereafter, 10 mL of methanol was added to the vessel, and the hydrogen gas evolved. All the experiments were performed at temperatures of 30, 40, 45, 50, 55, and 60 °C. The catalytic stability test of the Cr_1.7_Ca_0.3_O_3_ catalyst was typically conducted in a batch reactor under controlled conditions. A specific amount of the catalyst was mixed with NaBH_4_, and methanol was then added. The reaction progress was monitored by measuring the hydrogen gas evolution rate. After each reaction cycle, the catalyst was separated from the reaction mixture via filtration. The regenerated catalyst was used for the next reaction cycle, and the process was repeated for 5 cycles.

## 3. Results and Discussion

**Figure 1:**
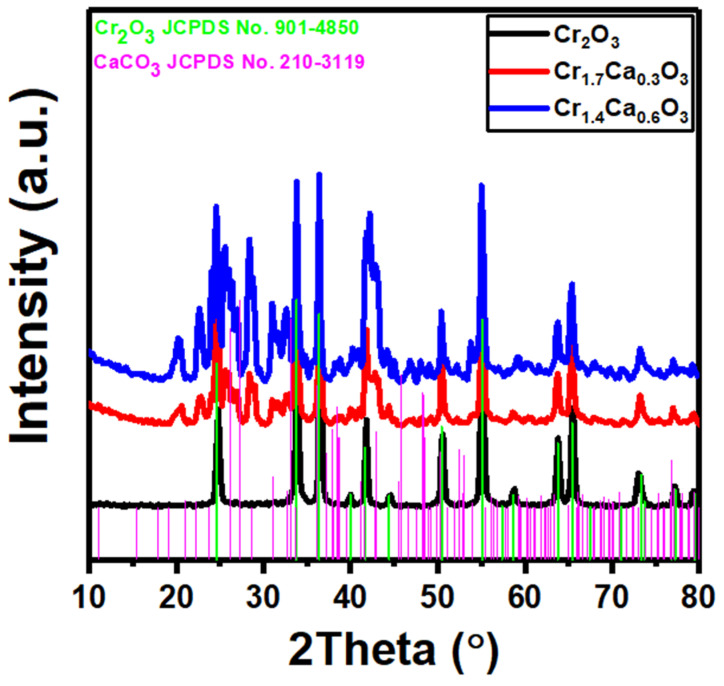
X-ray powder diffraction of Cr_2−x_Ca_x_O_3_ nanoparticles.

To determine the crystallinity of the studied samples, X-ray diffraction (XRD) was performed for the Cr_2−x_Ca_x_O_3_ nanoparticles_._ Figure 1 represents the XRD data of the Cr_2_O_3_ at different doping ratios of calcium. The obtained XRD pattern corresponds to chromium oxide (Cr_2_O_3_), as shown in the black curve in Figure 1, where it is indexed using JCPDS No. 901-4850. Therefore, the peaks for Cr_2_O_3_ observed at 2theta of 24.8, 33.7, 36.2, 41.7, 50.7, 55.13, 63.7, and 65.5°. These peaks can be assigned to the (012), (104), (110), (113), (024), (116), (214), and (300) planes of the Cr_2_O_3_ rhombohedral phase [70,71]. The additional peaks in the red and blue patterns belong to the calcium carbonate CaCO_3_, and they match with the standard JCPDS No. 210-3119. Moreover, the rate of carbonation rises with an increasing surface area due to the rapid reaction of CaCO_3_ [72,73]. Accordingly, XRD analysis confirms the formation of a Cr_2_O_3_/CaCO_3_ nanocomposite catalyst. The estimated lattice constant values for Cr_2_O_3_, Cr_1.7_Ca_0.3_O_3_, and Cr_1.4_Ca_0.6_O_3_ were a = 4.9132, 4.92572, and 4.9365 Å and c = 13.4993, 13.5005, and 13.4979 Å. The observed trends could be attributed to the difference in the ionic radii between chromium (Cr^3+^) and calcium (Ca^2+^). As calcium replaces chromium in the lattice, the larger sizes of the Ca^2+^ ions cause the surrounding lattice to expand.

By using the Scherrer equation ($D=\frac{0.9\lambda }{\beta cos\theta }$) [74], the average crystal size (D) of the three main peaks (33.7, 36.2, 55.13°) was calculated to be 25 nm.

**Figure 2:**
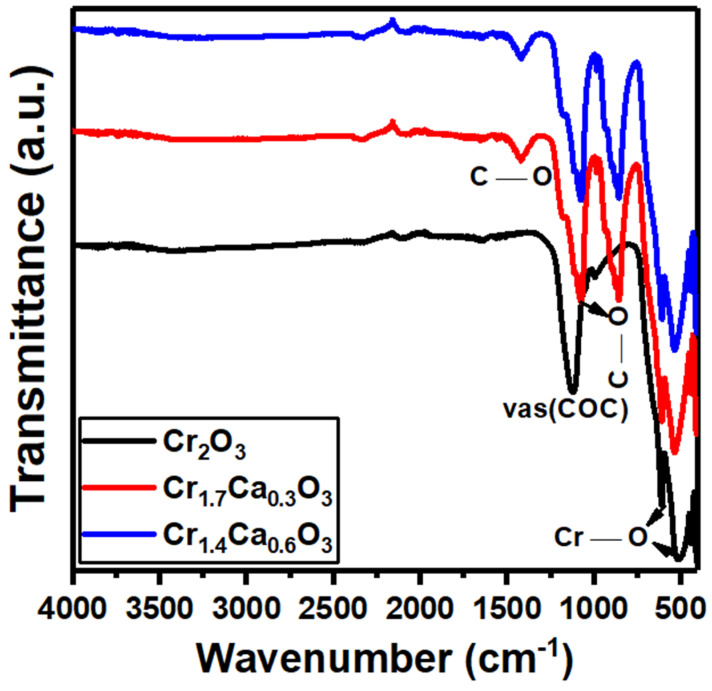
FT-IR spectra of Cr_2−x_Ca_x_O_3_ nanoparticles.

The FTIR measurements in Figure 2 were performed for the Cr_2−x_Ca_x_O_3_ nanoparticles. The noted bands at 435, 550, 620, and 1092 cm^−1^ correspond to the chromium oxide (Cr_2_O_3_). The high intensity of the peaks of the Cr_2_O_3_ bands is evidence of the crystalline nature of the sample. The two peaks that appeared at 550 and 620 cm^−1^ belong to the Cr–O bond stretching modes [75,76]. The band at 1120 cm^−1^ is based on the νas(COC) vibrations in the Cr_2_O_3_ lattice [77]. With the growth of the CaCO_3_, the peaks at 1420 and 860 cm^−1^ belong to the C–O asymmetrical and symmetrical vibrations [78]. Also, the bands at 450 correspond to Ca–O metal oxide [79]. Finally, the FTIR peak at 1068 cm^−1^ of CaCO_3_ is attributed to the in-plane bending vibration of the C-O bond in the carbonate ions (CO_3_^2−^) [80]. All these findings reveal the formation of a Cr_2_O_3_/CaCO_3_ nanocomposite catalyst.

**Figure 3:**
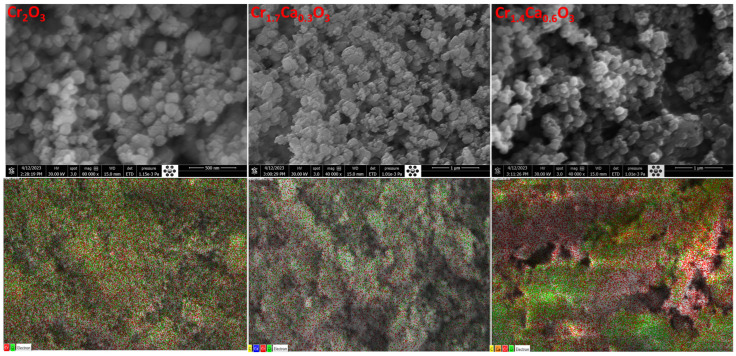
ESEM surface morphology analysis and elemental mapping for Cr_2−x_Ca_x_O_3_ nanoparticles.

Figure 3 shows the ESEM micrographs of the Cr_2_O_3_ at different doping ratios of calcium. The quasi-spherical shape of the Cr_2_O_3_ nanoparticles is detected, which may be beneficial for their application because this provides a high surface area, which is important for gas sensors, catalysts, and battery electrodes. The irregularities on the surface can provide a greater surface area for interactions with other molecules, which can enhance their catalytic activity and their ability to bind to biomolecules [81]. After the addition of calcium, the surface morphology is still quasi-spherical.

The elemental mapping was also used to study the interactions between the Cr_2_O_3_ and CaCO_3_ phases. Ca and O atoms are concentrated around the Cr_2_O_3_ particles, as shown in Figure 3. This suggests that there is a strong interaction between the two phases.

**Figure 4:**
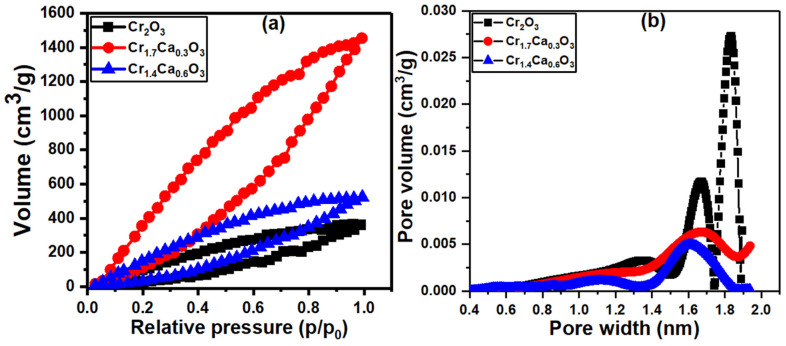
Plots of (**a**) nitrogen adsorption–desorption isotherm analysis and (**b**) pore size distribution for Cr_2−x_Ca_x_O_3_ nanoparticles.

BET surface area analysis is important for hydrogen catalysts because it provides information about the specific surface area of the catalyst material, which is related to its particle size and morphology. The specific surface area can have a fundamental influence on the characteristics and performance of the catalyst [82]. In this context, we generated N_2_ adsorption–desorption isotherms for the Cr_2−x_Ca_x_O_3_ nanoparticles, as shown in Figure 4a. The broadness of the curves is also related to the textural properties of the catalyst, such as its surface area and pore volume. A catalyst with a larger surface area and higher pore volume will generally have a broader adsorption–desorption curve. The samples Cr_2_O_3_, Cr_1.7_Ca_0.3_O_3_, and Cr_1.4_Ca_0.6_O_3_ showed BET surface areas of 116, 382, and 125 m^2^/g, respectively. The significant increase in surface area can be attributed to the formation of pores within the material. Ca atoms, being larger than Cr atoms, can disrupt the crystal structure of Cr_2_O_3_, leading to the formation of voids and channels. These pores contribute significantly to the overall surface area. From Figure 4b, the pore sizes estimated according to the BJH model were 1.88, 1.77, and 1.56 nm for Cr_2_O_3_, Cr_1.7_Ca_0.3_O_3_, and Cr_1.4_Ca_0.6_O_3_, respectively. The pore sizes reported (1.56–1.88 nm) fall within the range of mesopores (2–50 nm). This type of pore structure is often desirable for catalysts, as it allows for good accessibility of the reactant molecules to the active sites on the catalyst’s surface.

As the calcium content increases in the Cr_2−x_Ca_x_O_3_ system, the pore size decreases. This is due to the addition of calcium, which leads to the formation of different crystal phases in the Cr_2−x_Ca_x_O_3_ system, as confirmed using XRD analysis. These phases might possess distinct pore structures with varying sizes.

**Figure 5:**
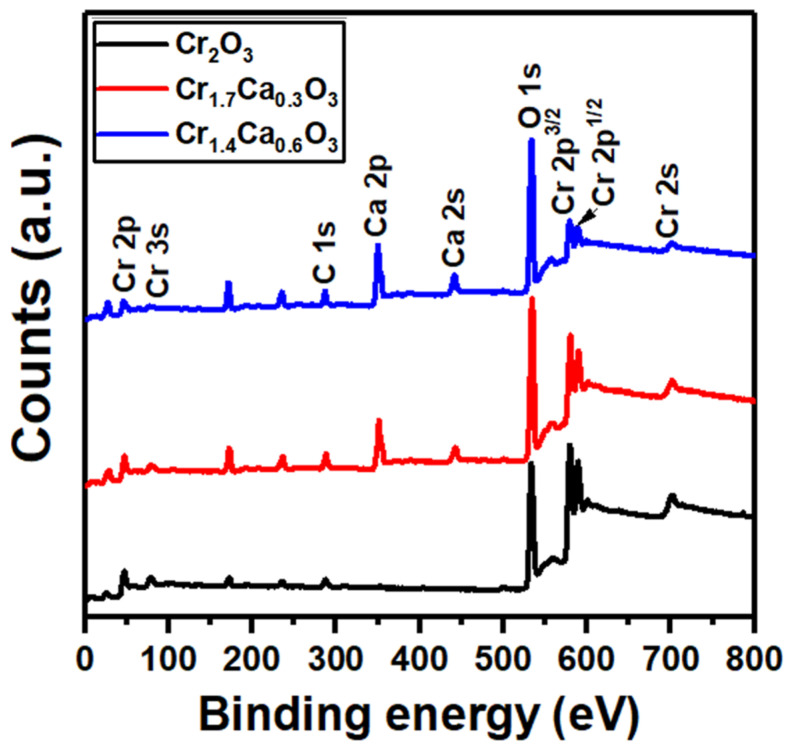
The XPS spectra analysis of Cr_2−x_Ca_x_O_3_ nanoparticles.

X-ray photoelectron spectroscopy (XPS) is a surface-sensitive analytical technique that can be used to study the chemical composition and electronic structure of nanoparticles. The XPS spectra of the Cr_2−x_Ca_x_O_3_ nanoparticles are plots of the intensity of the emitted electrons as a function of their kinetic energy (Figure 5). The different peaks in the XPS spectrum correspond to the Cr 2p, Ca 2p, and O 1s elements present in the nanoparticles. The elemental ratios of chromium to oxygen in the pure Cr_2_O_3_ sample were close to the expected ratios. The binding energies of Cr 2p^3/2^, 2p^1/2^, O 1s, C 1s, and Ca 2p are observed in Figure 5. Also, the carbon tape that was used to hold the sample within the chamber contributes to C 1s.

**Figure 6:**
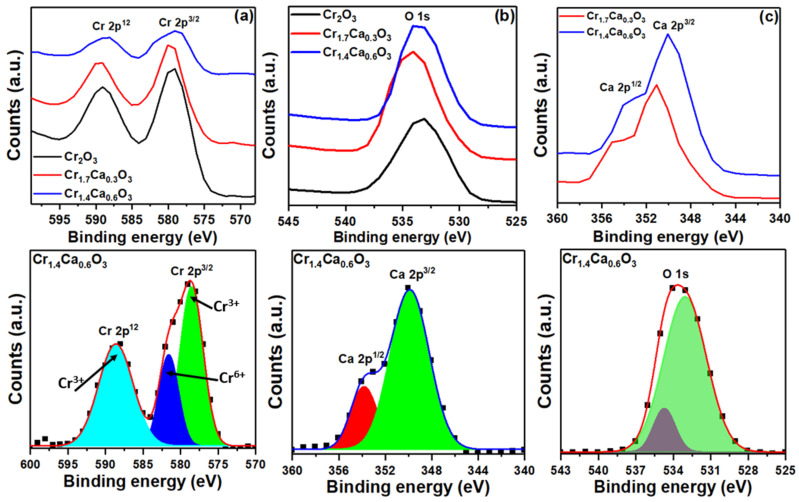
The XPS graphs of (**a**) Cr 2p, (**b**) O 1s, and (**c**) Ca 2p for Cr_2−x_Ca_x_O_3_ nanoparticles.

The XPS data for Cr 2p^3/2^ and 2p^1/2^, O 1s, and Ca 2p^3/2^ and 2p^1/2^ are plotted in Figure 6. The Cr 2p^3/2^ and 2p^1/2^ peaks are due to the core electrons of chromium and showed a separation of 10 eV. These results agree with the findings provided in the literature and demonstrate the development of Cr_2_O_3_ nanoparticles [83]. A shift to a higher binding energy in the Cr 2p XPS peaks typically indicates an increase in the oxidation state of the chromium. This can be due to the transfer of electrons from the Cr atom to neighboring atoms, leading to a more positive charge on the Cr atom. Higher oxidation states of Cr are beneficial for hydrogen production catalysts as they participate in the adsorption and activation of reactants. Two peaks are observed in the deconvoluted XPS spectra: a lower peak at 578 eV assigned to the Cr^3+^ state and a higher peak at 581 eV attributed to the Cr^6+^ state. Both peaks suggest the presence of chromium in multiple oxidation states on the sample surface. Although the bulk material is α-Cr_2_O_3_ (containing Cr^3+^), the surface appears partially oxidized to expose Cr^6+^ ions [84]. The O 1s peak at 533.08 eV is due to the core electrons of oxygen [85]. The O 1s peak can be deconvoluted into three peaks at 529.5, 531.1, and 532.8 eV. The binding energy of lattice oxygen (O^2−^) is typically around 529.5 eV. However, the peak cantered at 531.1 eV is typically associated with surface-adsorbed oxygen (O^−^). In addition, the binding energy of the O hydroxyl from O 1s in the XPS spectra is cantered at 532.8 eV [86].

The XPS peak position of calcium is typically a doublet, with two peaks at around 347.8 eV and 348.8 eV (see Figure 6c). The peak at 347.8 eV is due to the 2p^3/2^ electrons, and the peak at 348.8 eV is due to the 2p^1/2^ electrons [87]. Moreover, the intensity of the two peaks increased with an increasing calcium content. The binding energy of the Ca 2p electrons in CaCO_3_ is slightly higher than that of metallic Ca (around 346.7 eV) due to the presence of the carbonate group (CO_3_^2−^). This chemical shift is expected and confirms the presence of calcium in a carbonated environment. Moreover, the interaction between CaCO_3_ and Cr_2_O_3_ can lead to the formation of new surface species, such as carbonates or oxycarbonates. CaCO_3_ might also affect the concentration of the oxygen vacancies in the Cr_2_O_3_ lattice. Oxygen vacancies are missing oxygen atoms in the crystal structure, and they can act as electron donors. An increase in the oxygen vacancies can lead to a decrease in the binding energy of the remaining O atoms. Accordingly, the XPS data of the Cr_2−x_Ca_x_O_3_ nanoparticles confirmed the desired oxidation states.

**Figure 7:**
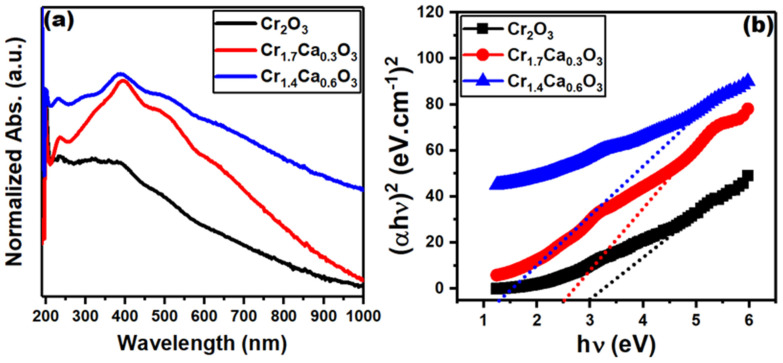
Graphs of (**a**) optical absorbance vs. wavelength and (**b**) (ahn)^2^ versus (hn) for Cr_2−x_Ca_x_O_3_ nanoparticles.

The optical properties of nanoparticles are significantly different from those of the same material in bulk form. This is because the optical properties of a material are determined by the interaction of light with its electrons. In nanoparticles, the electrons are confined to a much smaller space, which changes the way they interact with light. Moreover, the optical properties of nanoparticles can be affected by several factors, including their size, shape, composition, and surrounding environment. The UV-Vis optical absorption spectra of the Cr_2−x_Ca_x_O_3_ nanoparticles are plotted in Figure 7a. The most intense band is the band gap transition, which occurs at a wavelength of about 300 nm. The 400 nm peak corresponds to a transition from the ground state to the excited state, while the 600 nm peak corresponds to a transition from the excited state to the second excited state. These transitions are called d-d transitions because they involve electrons moving between the d orbitals of the Cr^3+^ ion [88]. The chromium 3 d orbitals split into two groups of orbitals when they are placed in an octahedral field. The three degenerate t_2g_ orbitals are oriented along the axes of the octahedron, while the two degenerate e_g_ orbitals are oriented between the axes. This splitting is caused by the interaction between the chromium 3 d electrons and the negative charges of the oxygen atoms in the octahedron. The two bands at 400 and 600 nm are associated with the ^4^A_2g_→^4^T_1g_ and ^4^A_2g_→^4^T_2g_ d^3^ intrinsic electronic transitions for Cr^3+^ ions in an octahedral field.

The addition of calcium to the Cr_2_O_3_ nanoparticles shifted the absorption edge to lower energies. The Tauc equation is a formula that can be used to determine the band gap of a semiconductor material from the optical absorption spectrum. The Tauc equation can be used to determine the band gap of a semiconductor material even if the absorption spectrum is not perfectly linear. This is because the equation is still valid in the region of the spectrum where the absorption coefficient is increasing rapidly [89].

(1) $$\alpha h\upsilon =A{\left(hv-E_{opt}\right)}^{0.5}$$

where *α* is the absorption coefficient, *h* is Planck’s constant, *ν* is the frequency of the light, A is a constant, and *E_opt_* is the band gap. The graphs of (α hv)^2^ versus (hv) shown in Figure 7b are linear in the region where the absorption coefficient is increasing rapidly. The band gap can then be estimated by extrapolating the linear part of the curve to the photon energy axis. The optical band gap of pure Cr_2_O_3_ nanoparticles is typically around 3.0 eV. Meanwhile, the addition of calcium reduced the band gap to 2.5 and 1.3 eV at 15.0 and 30.0% doping ratios.

The development of new and improved hydrogen evolution catalysts from NaBH_4_ is an active area of research. The goal is to develop catalysts that are more efficient, stable, and cost-effective. This would make hydrogen evolution catalysts from NaBH_4_ even more attractive for a wider range of applications. Sodium borohydride is a viable option for producing hydrogen. It is a simple, efficient, and environmentally friendly process. Hydrogen can be produced from the sodium borohydride reaction with methanol [90]:

NaBH_4_ + 4CH_3_OH → NaB(OCH_3_)_4_ + 4H_2_↑ + heat (2)

**Figure 8:**
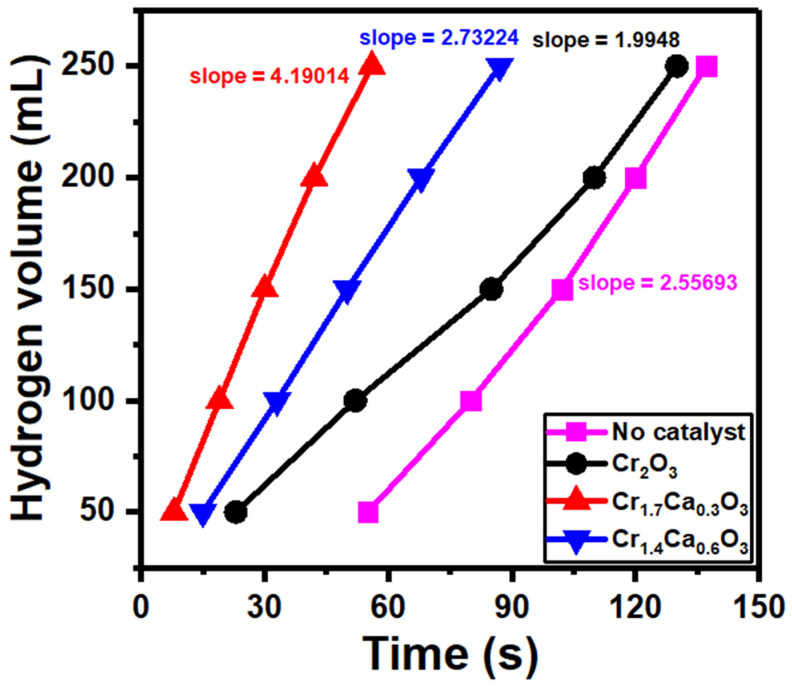
The volume of hydrogen vs. time produced at different catalytic Cr_2−x_Ca_x_O_3_ nanoparticles.

The methanolysis of sodium borohydride is a relatively simple and efficient process. The catalyst’s surface plays an important role in the methanolysis of the sodium borohydride reaction. It provides a surface onto which the BH_4_ ions can be adsorbed, and it facilitates the transfer of electrons from the BH_4_ ions to the catalyst’s surface [91]. This results in the formation of negatively charged H^−^ ions, which are the active species in the reaction. Figure 8 shows the volume of hydrogen produced at different catalytic Cr_2−x_Ca_x_O_3_ nanoparticles. The catalyst was used to speed up the reaction and increase the yield of hydrogen. The slope can also be used to calculate the rate constant of the reaction, which is a specific value that describes the inherent speed of the reaction under certain conditions. The catalyst Cr_1.7_Ca_0.3_O_3_ showed the highest slope (4.19014), indicating a faster reaction rate, meaning more hydrogen gas was produced per unit time.

The Cr-Ca-H complex is a strong reducing agent. It can donate an electron to the protonic hydrogen in CH_3_OH, which results in the formation of H_2_ gas. The boron atom in BH_3_OCH_3_ is electron-deficient. It can accept an electron from the CH_3_O group, which results in the formation of BH_3_OCH_3_ [92].

The hydrogen generation rate is a useful way to compare the catalytic performance of different catalysts. A catalyst with a higher hydrogen generation rate is a more efficient catalyst. The hydrogen volume (*V*), the mass of the catalyst (*m_cat_*), and time (*t*) are used to estimate the hydrogen generation rate (*r*) from the following equation [93]:

(3) $$r=\frac{V}{t\cdot m_{cat}}$$

**Figure 9:**
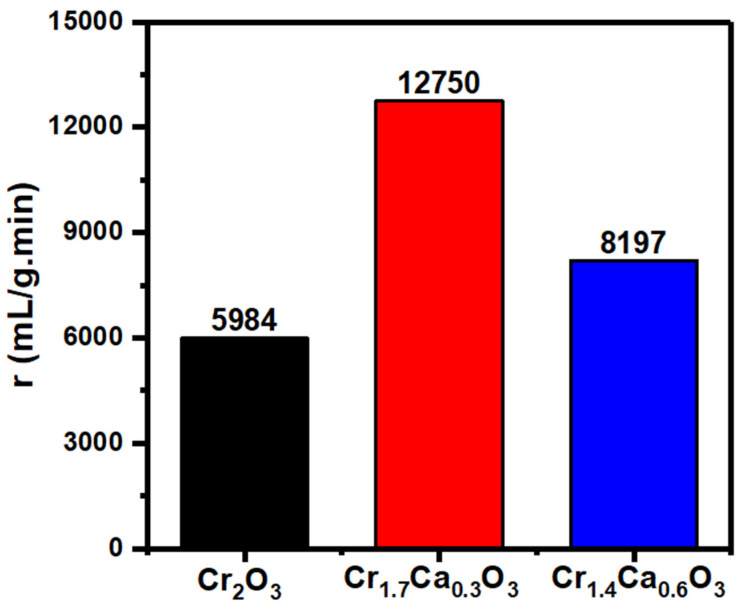
The generation rates of hydrogen for Cr_2−x_Ca_x_O_3_ catalysts.

Figure 9 compares the rates of hydrogen generation from NaBH_4_ methanolysis using different Cr_2−x_Ca_x_O_3_ catalysts. The slopes of the straight lines in Figure 8 were used to compute the values of the generation rates (*r*). The generation rate values were 5984, 12,750, and 8197 mL/g/min for Cr_2_O_3_, Cr_1.7_Ca_0.3_O_3_, and Cr_1.4_Ca_0.6_O_3_, respectively. The Cr_2_O_3_ catalyst has a relatively low surface area and limited porosity, restricting the number of active sites available for NaBH_4_ adsorption and reaction. This translates to a slower reaction rate and lower hydrogen production.

The sample with the composition Cr_1.7_Ca_0.3_O_3_ showed the highest generation rate (20,632 mL/g/min). The Cr_1.7_Ca_0.3_O_3_ catalyst is a good catalyst for the hydrogen evolution reaction because it has a high surface area and a high concentration of surface sites. Its high surface area allows the catalyst to adsorb a lot of BH_4_ ions, and the high concentration of surface sites allows the catalyst to activate the BH_4_ ions efficiently. Therefore, a large amount of hydrogen will be produced in a short time.

**Figure 10:**
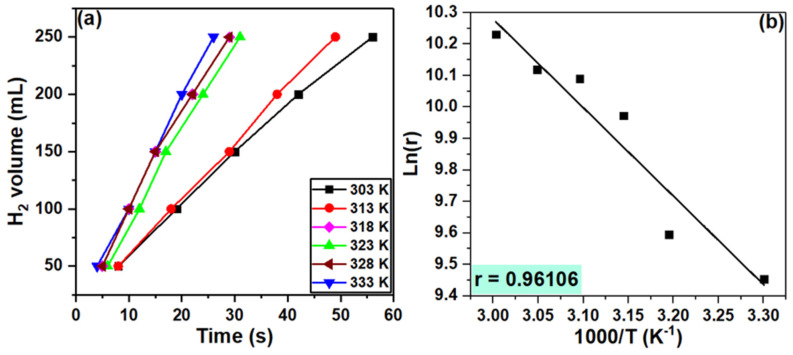
Plots of (**a**) H_2_ volume versus time at different temperatures and (**b**) ln(r) versus 1000/T for Cr_1.7_Ca_0.3_O_3_ catalyst.

The effect of temperature on the hydrogen volume as it evolved over time for the Cr_1.7_Ca_0.3_O_3_ catalyst is depicted in Figure 10a. Increasing the temperature typically has a positive effect on the volume of H_2_ produced over time. An increased temperature can enhance the activity of the catalyst by increasing the rate of the adsorption and desorption of reactants and products on the catalyst surface. This allows for faster reaction cycles and subsequently higher H_2_ production.

The activation energy explains the role of the catalyst in improving the catalytic methanol decomposition of sodium borohydride for hydrogen production. It represents the minimum energy barrier that reacting molecules must overcome for the reaction to proceed. The Arrhenius equation expresses the relationship between the hydrogen production rate (*r*) and the temperature (*T*) [94]:

(4) $$r=A\;exp(-E_{a}/(RT))$$

where *A* is the reaction constant, the activation energy is *E_a_*, and *R* is the gas constant (8.314 J/mol/K). The activation energy can be calculated from the slope of the ln(k) versus the 1000/T plot for the Cr_1.7_Ca_0.3_O_3_ catalyst given in Figure 10b. The resulting *E_a_* value was 23.37 kJ/mol. The activation energy of 23.37 kJ/mol suggests that the catalytic methanolysis of sodium borohydride requires a relatively low energy input to proceed. This is a positive finding, as it indicates that the reaction can be driven under mild conditions, potentially reducing the energy demands and cost of hydrogen production [95,96].

Table 1 provides a direct comparison of the efficiency of the Cr_1.7_Ca_0.3_O_3_ catalyst for the hydrogen evolution reaction with other catalysts. From this table, we can see that the Cr_1.7_Ca_0.3_O_3_ catalyst is superior to many catalytic materials for the hydrogen evolution reaction. It has a high hydrogen generation rate, is stable under reaction conditions, and is relatively inexpensive to produce. The high hydrogen generation rate of the Cr_1.7_Ca_0.3_O_3_ catalyst is attributed to the presence of calcium ions in the catalyst. The calcium ions play a role in activating the BH_4_ ions, which are the active species in the hydrogen evolution reaction. Accordingly, we suppose the wide application of this material as a promising catalyst for hydrogen production from sodium borohydride.

**Figure 11:**
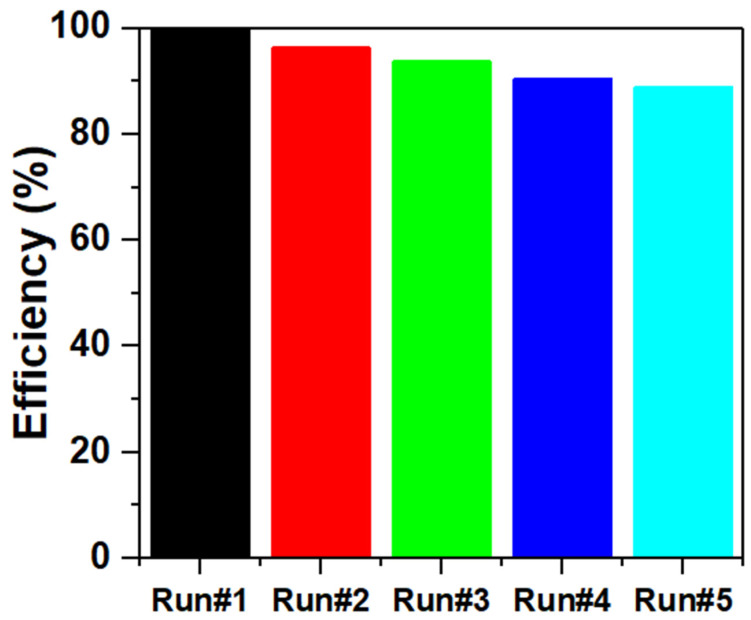
The generation rates of hydrogen for Cr_2−x_Ca_x_O_3_ catalyst.

In NaBH_4_ methanolysis, the reusability test assesses the stability and activity of the catalyst over multiple reaction cycles. This is crucial for practical applications, as a good catalyst should maintain its effectiveness without requiring frequent replacement. The Cr_1.7_Ca_0.3_O_3_ catalyst was subjected to NaBH_4_ methanolysis under the same conditions over five cycles. The results are compared in Figure 11 to the initial performance to determine the loss of activity or conversion after each cycle. The catalytic efficiency of the catalyst decreased to 88.9% after five cycles.

**Figure 12:**
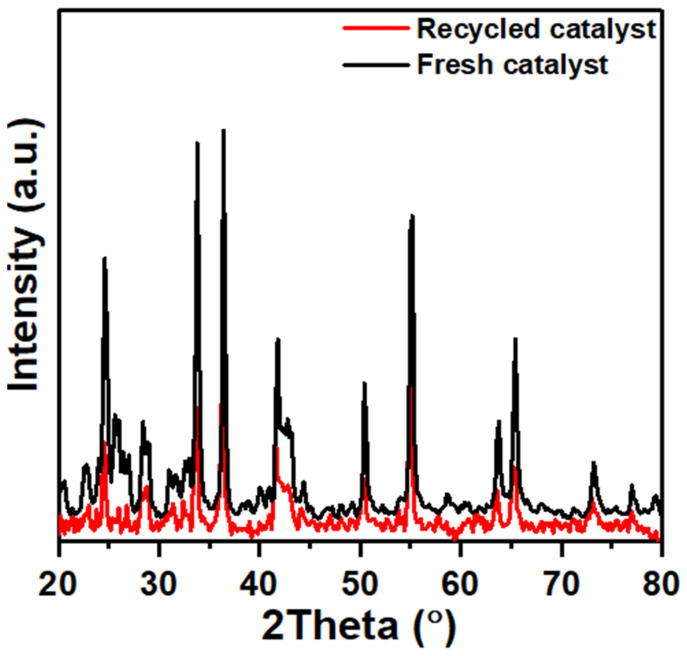
The XRD stability test for Cr_2−x_Ca_x_O_3_ catalyst.

Some metal ions from the Cr_2_O_3_ phase might leach into the solution during the reaction cycles. This loss of active metal can lead to a decrease in the overall catalytic activity. The CaCO_3_ support might also contribute to the leaching of calcium ions, potentially affecting the catalyst’s stability. The XRD stability test of the Cr_2−x_Ca_x_O_3_ catalyst used for NaBH_4_ methanolysis is a method to assess the crystallinity and structural integrity of the catalyst material after five reaction cycles. The XRD patterns obtained after five cycles are compared to the initial pattern, as shown in Figure 12.

The test reveals that the catalyst’s crystal structure remains intact. However, the changes in the peak intensity indicate a decrease in the concentration of active sites on the catalyst surface, potentially leading to activity loss.

## 4. Conclusions

Cr_2−x_Ca_x_O_3_ (x = 0, 0.3, and 0.6) nanocomposite catalysts were prepared successfully using sol-gel and calcination routes. XRD demonstrated the crystallinity of the Cr_2−x_Ca_x_O_3_ samples and provided an average crystallite size of 25 nm. The quasi-spherical shape of the Cr_2_O_3_ nanoparticles was detected in SEM images, which could be beneficial for their applications. The BET surface area values of Cr_2_O_3_, Cr_1.7_Ca_0.3_O_3_, and Cr_1.4_Ca_0.6_O_3_ were 116, 382, and 125 m^2^/g, respectively. The XPS data of the Cr_2−x_Ca_x_O_3_ nanoparticles confirmed the desired oxidation states. The optical band gap of pure Cr_2_O_3_ nanoparticles is typically around 3.0 eV. Meanwhile, the addition of calcium reduced the band gap to 2.5 and 1.3 eV at 15.0 and 30.0% doping ratios. The catalyst was used to speed up the reaction and increase the yield of hydrogen. The sample with the composition Cr_1.7_Ca_0.3_O_3_ showed the highest generation rate (12,750 mL/g/min). The Cr_1.7_Ca_0.3_O_3_ catalyst is superior to many catalytic materials for the hydrogen evolution reaction. Accordingly, we suppose the wide application of this material as a promising catalyst for hydrogen production from sodium borohydride.

## Figures and Tables

**Table 1:** Comparison of the Cr_1.7_Ca_0.3_O_3_ catalyst performance with other catalysts for hydrogen evolution.

Catalyst Material	Type	Hydrogen Generation Rate (mL/g/min)	Ref.
Cu_1.7_Ca_0.3_O/GO	Powder	9809	[93]
Co–Cr–B	Powder	2100	[94]
NiS-g-C_3_N_4_	Powder	8654	[97]
NiCr	Alloy	30,608	[98]
Cr_0.0125_-Ni-W-B	Powder	335	[99]
Fe_3_O_4_/FeS_2_/g-C_3_N_4_	Powder	8480	[100]
CoB/Ag–TiO_2_	Powder	393	[101]
Co-Cr-B/NG	Powder	2231.7	[102]
Co-CaCO_3_	Powder	6666	[103]
CuCo_2_O_4_	Powder	1370	[104]
Ni-Fe-B	Powder	2910	[105]
Co–Co_3_O_4_	Powder	2446	[106]
Cr_1.7_Ca_0.3_O_3_	Powder	12,750	This study

## Data Availability

Data will be made available on reasonable request.
